# Expression Patterns of Protein Kinases Correlate with Gene Architecture and Evolutionary Rates

**DOI:** 10.1371/journal.pone.0003599

**Published:** 2008-10-31

**Authors:** Aleksey Y. Ogurtsov, Leonardo Mariño-Ramírez, Gibbes R. Johnson, David Landsman, Svetlana A. Shabalina, Nikolay A. Spiridonov

**Affiliations:** 1 National Center for Biotechnology Information, National Library of Medicine, National Institutes of Health, Bethesda, Maryland, United States of America; 2 Division of Therapeutic Proteins, Center for Drug Evaluation and Research, U. S. Food and Drug Administration, Bethesda, Maryland, United States of America; University of Pennsylvania School of Medicine, United States of America

## Abstract

**Background:**

Protein kinase (PK) genes comprise the third largest superfamily that occupy ∼2% of the human genome. They encode regulatory enzymes that control a vast variety of cellular processes through phosphorylation of their protein substrates. Expression of PK genes is subject to complex transcriptional regulation which is not fully understood.

**Principal Findings:**

Our comparative analysis demonstrates that genomic organization of regulatory PK genes differs from organization of other protein coding genes. PK genes occupy larger genomic loci, have longer introns, spacer regions, and encode larger proteins. The primary transcript length of PK genes, similar to other protein coding genes, inversely correlates with gene expression level and expression breadth, which is likely due to the necessity to reduce metabolic costs of transcription for abundant messages. On average, PK genes evolve slower than other protein coding genes. Breadth of PK expression negatively correlates with rate of non-synonymous substitutions in protein coding regions. This rate is lower for high expression and ubiquitous PKs, relative to low expression PKs, and correlates with divergence in untranslated regions. Conversely, rate of silent mutations is uniform in different PK groups, indicating that differing rates of non-synonymous substitutions reflect variations in selective pressure. Brain and testis employ a considerable number of tissue-specific PKs, indicating high complexity of phosphorylation-dependent regulatory network in these organs. There are considerable differences in genomic organization between PKs up-regulated in the testis and brain. PK genes up-regulated in the highly proliferative testicular tissue are fast evolving and small, with short introns and transcribed regions. In contrast, genes up-regulated in the minimally proliferative nervous tissue carry long introns, extended transcribed regions, and evolve slowly.

**Conclusions/Significance:**

PK genomic architecture, the size of gene functional domains and evolutionary rates correlate with the pattern of gene expression. Structure and evolutionary divergence of tissue-specific PK genes is related to the proliferative activity of the tissue where these genes are predominantly expressed. Our data provide evidence that physiological requirements for transcription intensity, ubiquitous expression, and tissue-specific regulation shape gene structure and affect rates of evolution.

## Introduction

Phosphorylation of serine, threonine and tyrosine residues in substrate proteins by protein kinases (PKs) provides a fundamental mechanism for the control of cell division, growth and apoptosis, metabolic activity, adhesion and migration, and mediates cell responses upon environmental stimuli [1], [2], [3]. At the molecular level, phosphorylation-dephosphorylation allows fast and sensitive regulation of enzyme activity. It is also a major mechanism of transmembrane signal transduction and amplification in the branching network of intracellular PK cascades that ultimately control gene expression by phosphorylation of transcription factors. Phosphorylation of protein substrates creates binding sites for protein domains which recognize specific phosphorylated amino acid sequences, thereby mediating protein-protein interactions [3], [4].

The eukaryotic PK superfamily is subdivided into two broad groups of conventional and atypical kinases. Conventional PKs have been classified into eight families based on the structure and sequence similarities of their conserved eukaryotic catalytic domains. A smaller group of atypical PKs consists of several families that do not carry well conserved kinase domains. Still, many atypical protein kinases show evidence for enzyme activity. The number of PK genes in the animal genome progressively grows from lower to higher organisms, paralleling the evolutionary increase in the total number of genes and the complexity of organization. The protein kinase complement of the human genome (kinome) includes 518 predicted genes, comprising the third-largest gene superfamily [5]. Comparative analysis of the mouse genome performed by different research groups identified 540 to 561 candidate protein kinase genes [6], [7]. According to a more recent conservative estimate, the human and the mouse genomes contain 504 and 508 PK genes, correspondingly [8]. The majority of the human protein kinases have orthologs in the mouse, implying similar biological functions in both organisms. Some of these enzymes are restricted to or predominantly expressed in specialized tissues or cell types.

Expression of PK genes is subject to complex transcriptional control, which is not fully understood. Although orthologization and evolutionary conservation of PK protein sequences has been well established, little is known about evolutionary conservation and the function of non-coding DNA sequences of PK genes. Insights into the function of non-coding DNA can be gained from comparative analysis. According to estimations by different authors, fraction of selectively constrained non-coding DNA sequences in mammalian genomes represent from 3% (when highly conserved sequences alone are taken into account) to 10% or more (when weaker conservation is also considered) [9], [10]. Evolutionary conservation of non-coding DNA is controlled, at least in part, by negative selection and high interspecies homology of non-coding DNA sequences suggest their important regulatory function. For example, the vast majority of experimentally defined binding sites for the human skeletal muscle-specific transcription factors are confined to the most constrained orthologous sequences in the rodent genome [11]. Because patterns of gene regulation and the corresponding regulatory controls are often conserved between species, cross-species sequence comparison, so-called “phylogenetic footprinting”, may identify functional gene regulatory elements. Alignment algorithms based on interspecies sequence comparison have successfully been used to identify regulatory sites of genes expressed in the skeletal muscle [11] and endothelial tissue [12]. Here we employed a similar approach for identification of regulatory elements in non-coding regions of mammalian PK genes.

We analyzed 497 orthologous genomic loci of the human and mouse PK genes with the total length over 64 Mb, constituting about 2% of a mammalian genome. The goals of the present study were: *i)* evaluation of sequence conservation and evolutionary rates in non-transcribed, transcribed and translated regions of PK genes, *ii)* evaluation of the gene architecture and features of structural domains in differentially expressed genes, *iii)* identification of sequence elements and regulatory signals associated with transcription levels and message abundance, *iv)* evaluation of PK tissue expression patterns and sequence elements associated with tissue-specific gene expression. Here we present data on the relative expression levels and tissue-specific expression for the human kinome. We show that architecture of regulatory PK genes significantly differs from other protein coding genes and explore relationships between gene structure, evolutionary conservation, transcript levels and breadth of gene expression. We demonstrate that architecture of PK genomic loci correlates with the mode of gene expression and proliferative activity of the tissue where these genes are predominantly expressed. We describe evolutionarily conserved signals associated with transcript abundance and tissue-specific expression. Our results suggest that requirements for ubiquitous expression and tissue-specific regulation affect gene structure and impose selection pressure on the protein-coding and non-coding gene regions.

## Results

### Transcription levels and tissue-specific expression of PK genes

**Figure 1:**
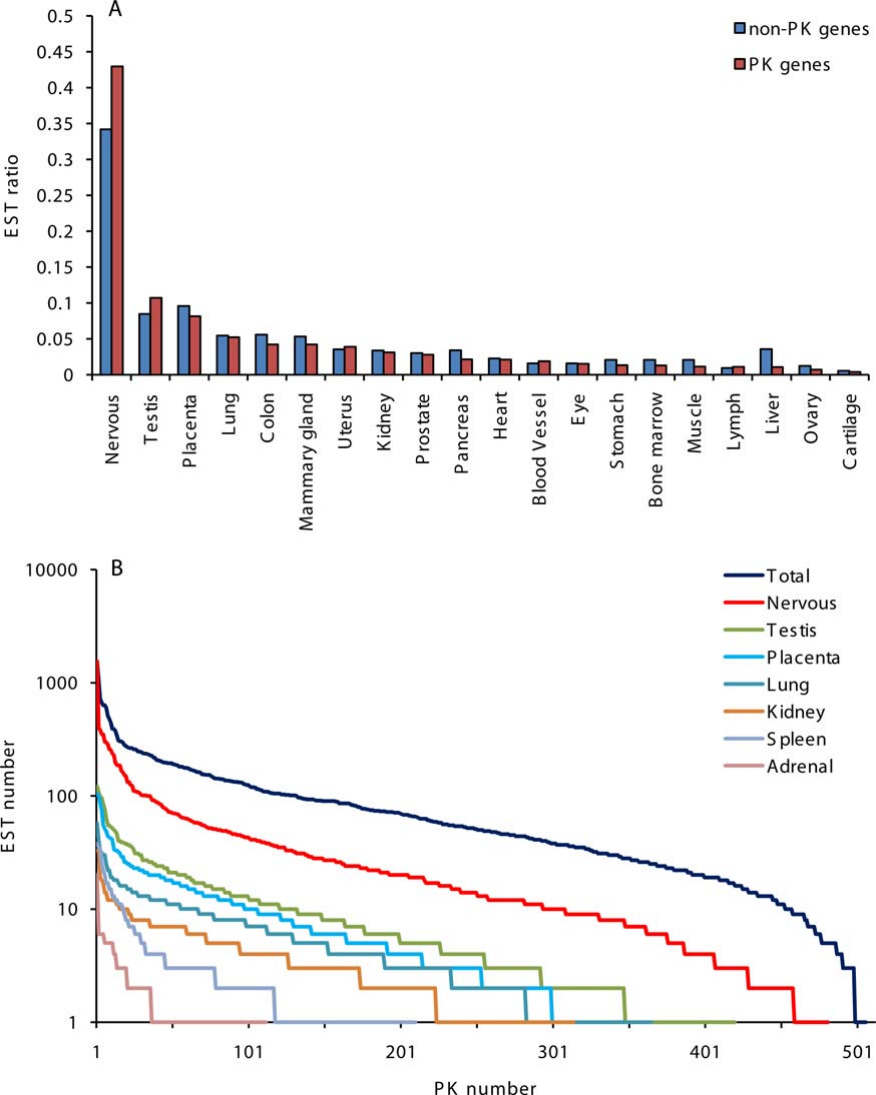
Relative tissue distribution and abundance of EST for 7,711 non-PK genes and 512 PK genes in GenBank, release 162. A. Relative tissue distribution of gene-specific EST for non-PK and PK genes. B. Abundance of PK-specific ESTs in libraries from normal human tissues. The data were graphed as EST number versus PK rank.

We evaluated expression of PK and non-PK genes based on the numbers of gene-specific ESTs in GenBank originating from normal human tissues, which reflect mRNA abundance and relative gene transcription levels. The vast majority of PK genes scored moderate EST numbers (84 ESTs average) in contrast with highly transcribed housekeeping non-PK genes (2000 or more ESTs). Based on this analysis, PKs fall into a category of moderately transcribed genes, which is consistent with their regulatory role. These data are in agreement with overall PK expression levels presented in the Gene Expression Atlas. For evaluation of PK expression patterns and the relative abundance of PK messages in different organs, we sorted gene-specific ESTs in accordance with their organ and tissue origins. ESTs originating from the brain and nervous tissue were most numerous in our dataset, followed by ESTs from testis and placenta (Figure 1). Distribution of PK-specific ESTs in 20 normal human organs and tissues is presented in Table S1. Our results showed that the majority of PK genes were broadly expressed in many tissues. At the same time, a number of PK genes showed distinct organ-specific and tissue-specific expression patterns.

Remarkably, EST libraries from nervous and testicular tissues were enriched with PK tags, relative to libraries from other tissues (Figure 1A), suggesting increased phosphorylation-dependent regulation in the brain and testis. Therefore, we focused our attention on PKs up-regulated in these two tissues. A diverse group of protein kinases that includes many members of CAMK1, CAMK2, DCAMKL, Eph, CDK, PKC and other families was predominantly expressed in the brain and nervous tissue (Table S1). Expression of VACAMKL, CaMK2 alpha, EphA7, PKC gamma and PAK5 was effectively restricted to the brain, and many of the nervous tissue-specific PKs scored high numbers of ESTs in GenBank, indicating active transcription. A smaller PK group was preferentially expressed in the testis (BRDT, HIPK4, MISR2, SgK307, SgK396, SSTK, TSSK1, TSSK2, TSSK4 and others). Several genes were predominantly expressed in placenta (TXK, FLT1, ACTR2B), muscle and heart (skMLCK, MSSK1), and other tissues. In the cases where experimental results are available, our identification of tissue-specific protein kinases was supported by data from literature. For example, five testicular protein kinases,TSSK1, TSSK2, SSTK, CAMK4, and Haspin, are specifically expressed in haploid germ cells and two of these enzymes (CAMK4 and SSTK) are indispensable for normal progression of spermatogenesis and male fertility [13], [14], [15], [16]. Experimental evidence for brain- and neuron-specific expression was obtained for VACAMKL [17], PAK5 [18], Eph receptor tyrosine kinase EphA4 [19], CaMK1 gamma [20], CaMK2 alpha [21], PKC gamma [22], CDK5 [23] and some other kinases identified in our search.

### Structural features of PK genes associated with expression levels and breadth

**Figure 2:**
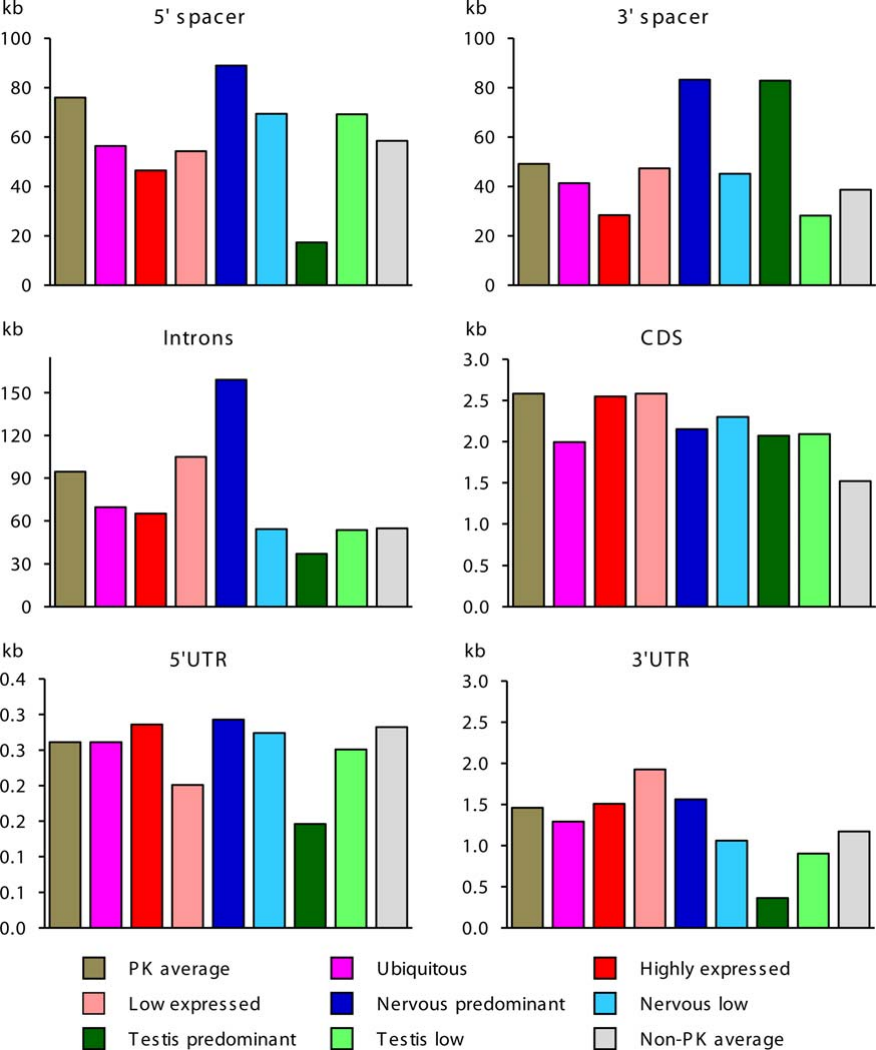
The length of the structural domains in human PK and non-PK genes. The following groups of differentially expressed PK genes were analyzed: all PK genes, high expression genes (75 genes with highest EST numbers), low expression genes (75 genes with lowest EST numbers), ubiquitously expressed genes, genes up-regulated in the nervous or testicular tissue, genes down-regulated in the nervous or testicular tissue. Sequence conservation was evaluated relative to mouse gene orthologs. CDS of extremely large PK titin was excluded from this analysis. Data are presented as averages.

For evaluation of gene architecture, we analyzed length and GC content in different gene functional domains in 510 human PK genomic loci. To compare structural properties of PK genes with overall trends for other genes, we used control group of 7,711 well-annotated human non-PK genes. Genomic architecture of PK and non-PK genes significantly differed. As seen from Figure 2, PK genes occupy larger genomic loci, possess significantly longer exons and spacer regions, and encode larger proteins, relative to the group of non-PK genes. PK genes also tend to have more GC-rich UTRs relative to non-PK genes (Table 1). Remarkably, lengths of gene loci, 5′-spacers, introns and UTRs of the human PK genes were ∼15% longer than for the mouse PK genes, revealing higher gene complexity (Figure S1). Same trend was observed for non-PK genes (data not shown).

**Table 1:** GC-content and human-mouse sequence conservation in structural domains of human PK and non-PK genes.

Features	Non- PK genes	PK genes	PK expression level		PK expression breadth				
					Ubiqui-tous	Nervous tissue		Testis	
			High	Low		Up-reg	Down-reg	Up-reg	Down-reg
**5′-spacer**									
Conservation, %	56.48	55.28	53.62	55.52	55.66	56.59	53.40	58.33	56.60
G+C content, %	45.53	47.39	47.33	47.36	50.65	49.17	49.54	48.05	49.82
**Promoter region**									
Conservation, %	59.97	60.02	58.26	59.63	61.38	61.17	55.96	60.88	60.37
G+C content, %	48.42	50.83	50.25	49.61	56.27	53.19	52.00	48.52	53.10
**Primary transcript**									
Conservation, %	59.88	59.03	59.70	59.28	59.17	58.41	57.78	66.54	61.46
G+C content, %	45.08	45.92	46.65	46.99	49.28	46.92	48.27	46.68	49.65
**5′UTR**									
Conservation, %	72.43	73.54	74.2	70.32	76.61	76.76	68.3	71.66	74.77
G+C content, %	61.27	65.32	66.58	61.01	72.39	64.3	67.12	60.49	67.05
**Protein coding regions**									
Conservation, %	85.28	87.08	87.81	84.85	88.80	88.57	85.03	84.83	87.40
G+C content, %	51.96	52.20	52.53	52.65	54.37	54.05	53.68	51.23	54.25
**Introns**									
Conservation, %	56.14	55.03	55.04	54.77	55.05	55.59	53.44	56.10	57.13
G+C content, %	43.64	45.08	45.82	45.96	48.63	46.53	47.40	40.92	48.97
Intron number	9.3	16.3	17	16.8	13.4	18	14	4.5	14.3
**3′UTR**									
Conservation, %	68.59	69.48	70.83	64.13	71.69	71.19	64.96	66.19	68.47
G+C content, %	42.90	45.20	46.12	46.43	47.39	46.55	46.55	41.44	47.06
**3′-spacer**									
Conservation, %	58.15	58.59	59.52	58.04	57.69	59.87	56.12	56.82	59.40
G+C content, %	44.11	46.44	47.73	46.77	48.87	47.22	48.34	44.33	46.86

Genomic repetitive elements were excluded from computation of sequence conservation. Data are presented as averages. Gene groups are defined in Figure 2 legend.

To analyze gene structural features associated with transcription levels, we selected groups of high and low transcribed PK genes. For both groups, we analyzed length, GC-content, and human-mouse sequence conservation in gene functional domains. The proximal 3 kb spacer regions immediately upstream from the translation start site that harbor promoters and the majority of known transcription factor sites in humans were analyzed separately. Results of this analysis are presented in Table 1 and Figure 2. Several structural features were associated with active transcription and elevated mRNA levels. Primary transcripts and introns of high expression genes were significantly shorter than primary transcripts and introns of low expression PK genes (*p*<0.05). Consistent with published data [24], this trend was also observed for non-PK genes. High expression PK genes also possessed longer (p<0.03) and a more conserved (*p*<0.02) 5′UTRs with significantly higher GC-content, and significantly more conserved 3′UTRs (*p*<10^−4^) with extended footprints, relative to low expression genes. We found no association between expression levels and the length of mature mRNA, the size of the protein and GC content in distant spacers, introns, and primary transcripts. Similar results were obtained for the mouse PK genes (Figure S1 and data not shown).

**Figure 3:**
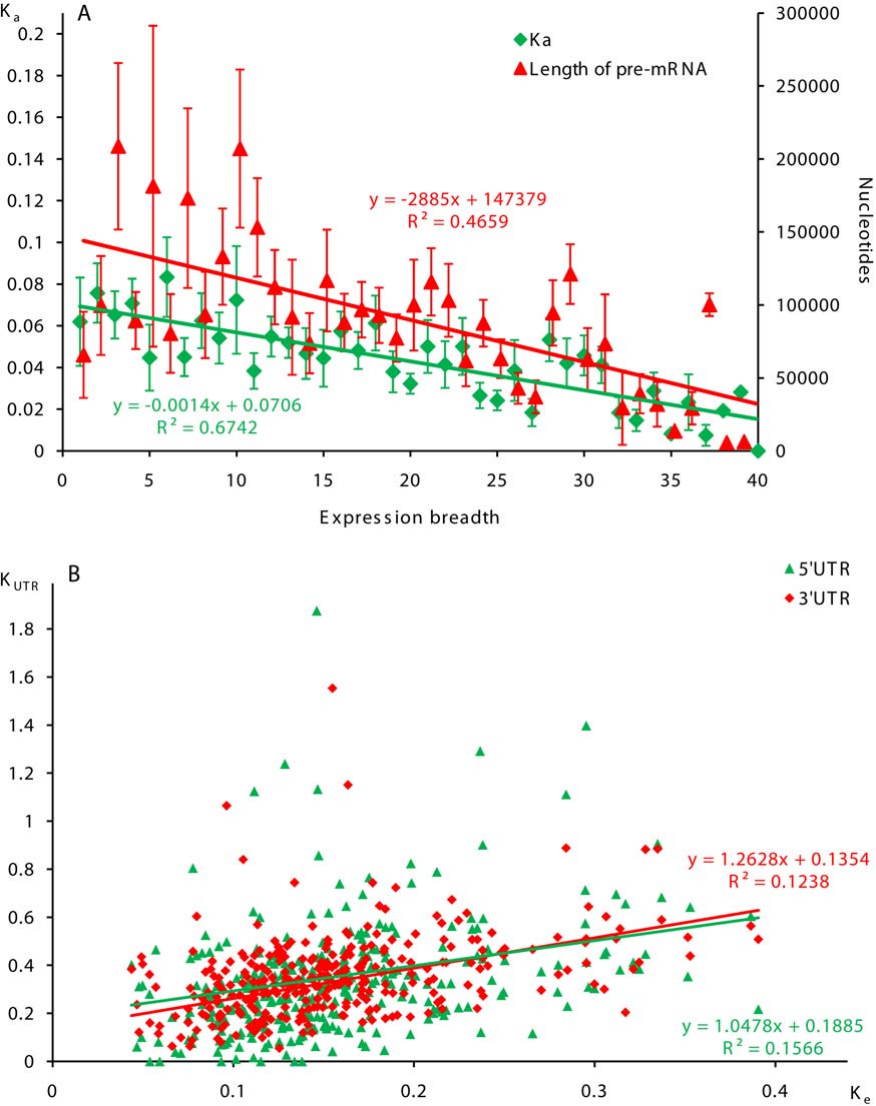
Correlations between the level of human-mouse evolutionarily divergence in protein coding and untranslated regions of human PK genes, expression breadth, and the size of pre-mRNA. A. Correlation between non-synonymous divergence, PK expression breadth and the size of pre-mRNA. Breadth of gene expression was estimated as the number of organ and tissue sources of gene-specific ESTs. Data are presented as averages and SEM. B. Correlations between evolutionarily divergence in protein coding regions and UTRs of PK genes.

We analyzed structural features of PK genes associated with breadth of expression (defined as the number of tissues where a gene is expressed). We observed strong negative correlation between the size of pre-mRNA and the number of expressing tissues (*R* = −0.67, *p* = 1.22×10^−6^, Figure 3A). We also found similar correlation for non-PK genes, which was primarily due to smaller size of introns in broadly expressed genes.

### Characteristic features of PK genes associated with expression in brain and testis

Distribution of ESTs in tissue libraries (Figure 1A) and expression profiling (Table S1) suggest that the brain and testis possess more complex phosphorylation dependent regulatory networks, relative to other organs. To identify gene structural features associated with expression in the nervous and testicular tissue, we analyzed non-overlapping groups of genes predominantly expressed in these tissues. PK genes up-regulated in the brain and testis were compared to control groups of ubiquitously expressed PK genes, and genes down-regulated in these organs. Overall gene organization and features of functional domains significantly differed between these groups (Table 1, Figure 2). Genomic loci and spacer regions of PK genes up-regulated in the nervous tissue were generally longer than those of ubiquitously expressed PKs (*p*<0.0005) and other analyzed PK groups. Similarly, primary transcripts and introns of PK up-regulated in the nervous tissue were dramatically longer than those of ubiquitously expressed PK genes (*p*<0.0004 and *p*<0.0005, correspondingly) and PK genes of other groups.

In contrast, genes up-regulated in the testis were significantly more compact than ubiquitously expressed PK genes (*p*<0.05) and genes predominantly expressed in nervous tissue (*p*<0.005), with shorter transcribed regions and smaller number of introns. Testicular PK genes had two to three times shorter 5′-spacers (*p*<0.005) with significantly lower GC content (*p*<0.02) in the promoter regions than ubiquitously expressed PKs genes and genes up-regulated in nervous tissue (Table 1, Figure 2). Testis-specific PK transcripts carried the shortest and least conserved UTRs among all analyzed groups of PK transcripts.

### Evolutionary divergence of the human and mouse PK genes

For evaluation of evolutionary divergence, we constructed detailed alignments for human and mouse PK genomic loci. Here we present data for 497 orthologous gene pairs that yielded complete collinear alignments of the transcribed regions, 5′- and 3′-spacer regions, collectively covering over 64 Mb of the human genome. Incomplete alignments that missed spacer regions due to deletions or genomic translocations were not used in our analysis. To compare evolutionary divergence of PK genes with overall trends for other genes, we constructed alignments for control group of 7,711 well annotated orthologous human and mouse non-PK genes.

Protein coding regions of the human and mouse PK orthologs were highly conserved (over 80% identity in nucleotide sequences). To evaluate selection pressure on coding sequences, we calculated levels of non-synonymous (K_a_) and synonymous (K_s_) human-mouse nucleotide substitutions in the protein coding regions of PK and non-PK genes using Yang's model [25]. Results of these calculations are presented in Table 2. The Wilcoxon rank sum test showed that the average K_a_ values and K_a_/K_s_ ratios were significantly lower for PK coding regions, relative to non-PK genes, indicating stronger purifying selection on PK amino acid sequences. Evolutionary changes in PK protein-coding regions were not homogeneous. Kinase modular domains in CDSs were more conserved than inter-domain regions. Within the group of PK genes, selective pressure on non-synonymous sites varied significantly depending on expression levels and the number of tissues in which genes were expressed. The level of non-synonymous substitutions in PK genes negatively correlated with breadth of gene expression (*R* = −0.82, *p* = 1.73×10^−9^, Figure 3A), which is consistent with a general trend for non-PK genes and correlations observed in protein coding regions of non-regulatory genes [26], [27]. The level of non-synonymous substitutions in PK genes also negatively correlated with gene expression levels (*R* = −0.72, *p* = 7.35×10^−8^, Figure S2) and positively correlated with the size of pre-mRNA (*R* = 0.39, *p*<0.01).

**Table 2:** Human-mouse evolutionary divergence in the protein coding and untranslated regions of domains of human PK and non-PK genes.

K values	Non- PK genes	PK genes	PK expression level		PK expression breadth				
					Ubiqui-tous	Nervous tissue		Testis	
			High	Low		Up-reg	Down-reg	Up-reg	Down-reg
**5′-spacer**									
**K_5′_**	0.346 (0.002)	0.339 (0.013)	0.322 (0.024)	0.356 (0.034)	0.298 (0.026)	0.288 (0.041)	0.396 (0.039)	0.325 (0.047)	0.340 (0.035)
**Protein coding regions**									
**K_e_**	0.171 (0.001)	0.159 (0.003)	0.145 (0.006)	0.186 (0.009)	0.135 (0.006)	0.149 (0.008)	0.177 (0.010)	0.189 (0.013)	0.144 (0.011)
**K_a_**	0.073 (0.001)	0.050 (0.002)	0.042 (0.005)	0.060 (0.005)	0.029 (0.003)	0.031 (0.012)	0.057 (0.008)	0.095 (0.012)	0.046 (0.009)
**K_s_**	0.558 (0.002)	0.548 (0.007)	0.540 (0.017)	0.560 (0.018)	0.530 (0.020)	0.538 (0.026)	0.609 (0.025)	0.510 (0.042)	0.540 (0.017)
**K_a_/K_s_**	0.128	0.089	0.080	0.110	0.050	0.090	0.090	0.190	0.080
**3′UTR**									
**K_3′_**	0.379 (0.002)	0.376 (0.012)	0.371 (0.038)	0.431 (0.018)	0.322 (0.022)	0.357 (0.028)	0.411 (0.023)	0.447 (0.047)	0.379 (0.021)
**Introns**									
**K_i_**	0.545 (0.001)	0.564 (0.006)	0.556 (0.013)	0.571 (0.014)	0.579 (0.020)	0.533 (0.013)	0.605 (0.021)	0.571 (0.046)	0.508 (0.016)

Evolutionary divergence in the protein coding regions (K_e_), 5′UTRs (K_5′_), and 3′UTRs (K_3′_) was calculated using Kimura's two parameter model [28]. Rates of synonymous (K_s_) and non-synonymous (K_a_) divergence were calculated using Yang's model [25]. Gene groups are defined in Figure 2 legend. Data are presented as averages and the standard error of the mean (SEM, shown in the parentheses).

On average, protein coding sequences of ubiquitous PKs evolved slower than those of differentially expressed PKs, as seen from their low K_a_ values (Table 2). PKs with restricted tissue expression (rank of expression breadth≤5) evolved significantly faster (*p*<0.001) than broadly expressed genes (rank of expression breadth>30). However, the group of PKs with restricted tissue expression is evolutionarily diverse, as seen from the high values of K_a_ standard errors for these kinases (Figure 3A), indicating strong variability in rates of their evolution. For example, PKs up-regulated in the highly proliferative testicular tissue evolved almost three times faster than PKs up-regulated in the minimally proliferative nervous tissue which evolved slow, similar to broadly expressed PKs, as seen from their low K_a_ values. Contrarily, K_s_ values in the protein coding regions did not differ significantly between ubiquitously and differentially expressed PK groups and did not correlate with gene expression patterns (Table 2), indicating similar levels of synonymous mutations. These results indicate that the differences in K_a_ values observed between the groups of ubiquitously and differentially expressed PKs are not caused by regional variations in the neutral mutation rate and reflect increased selective pressure on amino acid sequences.

Interestingly, PKs down-regulated in the nervous tissue and PKs with generally low transcription levels also displayed increased divergence in amino acid sequences. Same trends were observed in 5′ and 3′UTRs.

**Figure 4:**
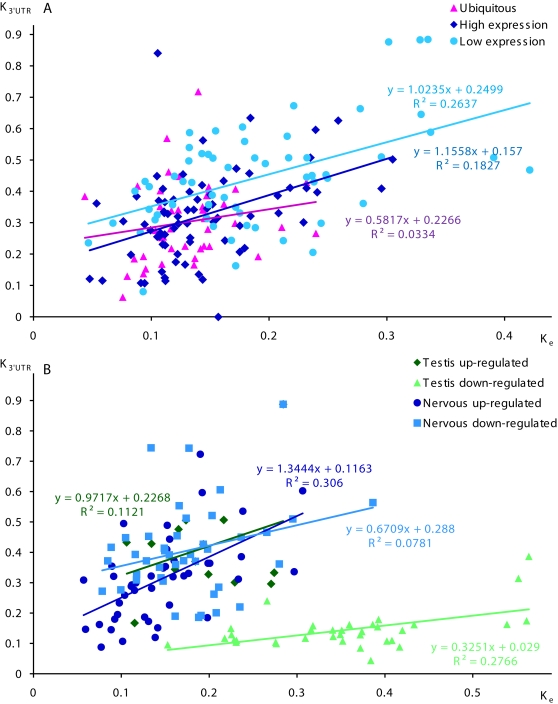
Correlations between the level of human-mouse evolutionarily divergence in protein coding regions and 3′UTRs of ubiquitous and differentially expressed human PK genes. A. Correlations between evolutionarily divergence in protein coding regions and 3′UTRs of high, low, and ubiquitously expression PK genes. B. Correlations between evolutionarily divergence in protein coding regions and 3′UTRs of PK genes up-regulated and down-regulated in nervous and testicular tissues.

We compared evolutionary rates in transcribed domains of PK and non-PK genes by evaluating the human-mouse evolutionary divergence in 5′UTRs (K_5′_), CDSs (K_e_), introns (K_i_), and 3′UTRs (K_3′_) using Kimura's two parameter model [28]. PK genes are characterized with lower K_e_ values, relative to non-PK genes, reflecting higher constraint on amino acid sequences. We also observed increased K_i_ values in PK introns, as compared to non-PK introns. As shown in Table 2, evolutionary divergence was significantly lower for both PK and non-PK genes in 5′UTRs (*p*<10^−7^) and 3′UTRs (*p*<10^−5^), relative to introns. We found significant positive correlations between levels of evolutionary divergence in CDSs and 3′UTR, in CDSs and 5′UTRs of PK genes (Figure 3B). Similar to K_a_ values, K_3′_ values inversely correlated with breadth of gene expression (*R* = −0.11, *p*<0.01). Positive correlation between K_e_ and K_3′_ values was observed for PKs predominantly expressed in nervous tissue, and for other differentially expressed PK groups (Figure 4). This trend was also observed for slow evolving ubiquitous PKs.

### Regulatory signals in PK genes associated with transcription levels

Taking into consideration strong relationships between gene transcription levels, evolutionary conservation, and the structure of regulatory domains, we attempted to identify evolutionary conserved DNA elements that regulate gene expression. For regulatory elements associated with transcript abundance, we searched for motifs over-represented in conserved promoter regions of high expression PK genes using the discriminating matrix emulator (DME) program. Conserved promoter sequences of low expression PK genes were used as a background set in this analysis. DME search revealed a number of motifs ranging from 6 to 10 nt which were significantly over-represented in promoters of high expression PK genes. Some of the characteristic over-represented motifs are shown on Figure S3, and the top 50 over-represented motifs are presented in Table S1. Promoters of high expression, actively transcribed PK genes were enriched with GC-rich motifs. In contrast, promoter regions of low expression PK genes were dominated by AT-rich low complexity motifs (data not shown). To identify potential binding sites for transcription factors, we searched through TRANSFAC database for position frequency matrices that match the motifs found by DME. Most common motifs were identified as conserved and degenerate binding sites for transcription stimulating proteins Sp1 and Sp3, core binding sequences for activator protein AP-2, characteristic for increased promoter performance, and other transcription factors. Some of the over-represented motifs were not identified as recognition sites for known DNA-binding proteins.

To identify sites in 5′UTRs associated with transcript abundance, we performed a search for evolutionarily conserved over-represented sequence elements using the SiteBD program. 5′UTRs of abundant transcripts have significantly higher GC-content and were enriched three-fold with repetitive GGCGGCGGC motifs (*p*<5×10^−55^), complementary CCGCCGCCG motifs (*p*<9×10^−39^), and other GC-containing sites, as compared to rare transcripts (Table S1). Several motif variants differing by nucleotide shifts were found. Unlike typical transcription factor binding sites, these motifs possessed a low degree of degeneration, mostly resided in 5′UTRs and were rarely encountered in the proximal spacers regions.

**Figure 5:**
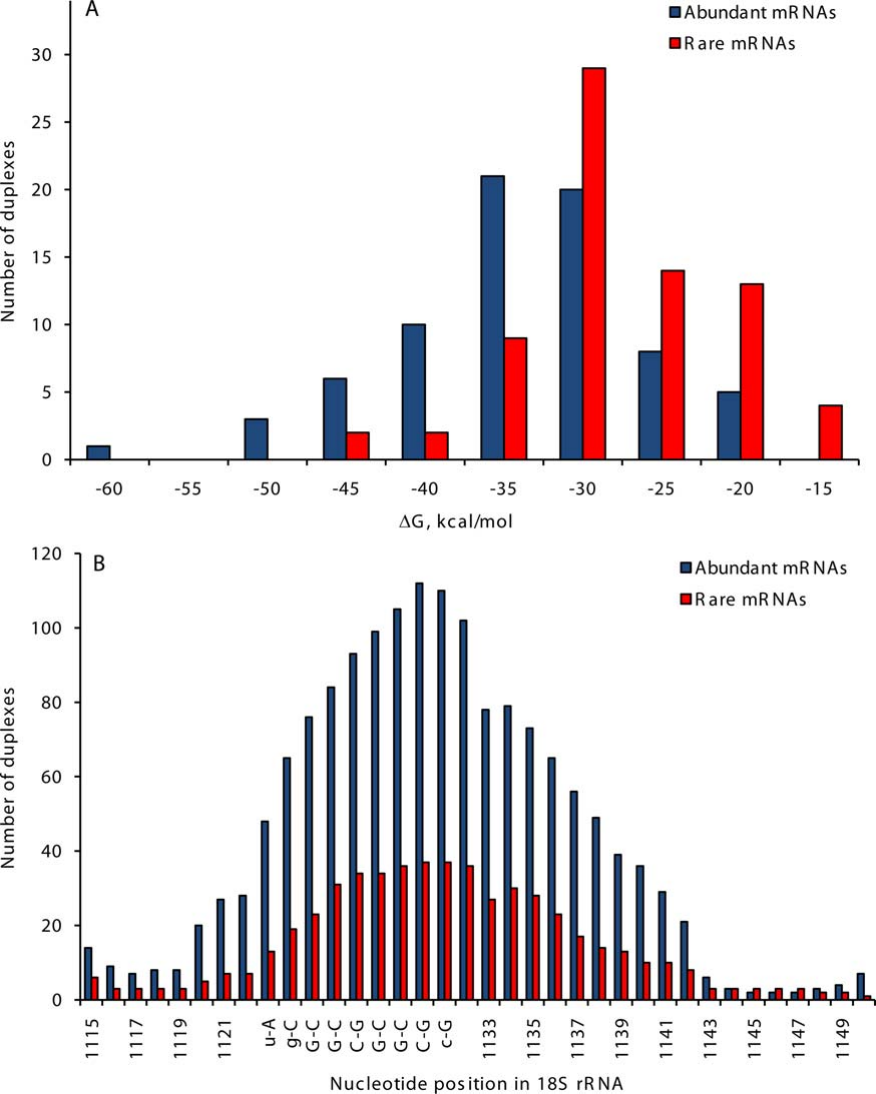
Hybridization affinity of 5′UTRs of PK genes to human 18S ribosomal RNA. A. Distribution of calculated energy of predicted duplex formation (ΔG) between human 18S rRNA and 5′UTRs of abundant and rare PK transcripts. Site of duplex formation between experimentally confirmed 18S rRNA “clinger” element and a core of 5′UTR translation enhancer [29] is shown. B. Hybridization affinity of abundant and rare PK transcripts to experimentally verified 18S rRNA “clinger” element [29].

To identify sites of potential interaction with ribosomes, we evaluated hybridization affinity of abundant and rare PK transcripts to 18S ribosomal RNA. As seen from Figure 5A, 5′UTRs of abundant transcripts possessed two to three-fold higher potential to form intermolecular duplexes with 18S ribosomal RNA, relative to 5′UTRs of rare PK transcripts. This effect was observed for theoretically predicted 18S rRNA “clinger” sites (data not shown), and also for an experimentally confirmed “clinger” element [29], which base pairs to a core of the translation enhancer commonly occurring in the 5′UTR (Figure 5B).

**Figure 6:**
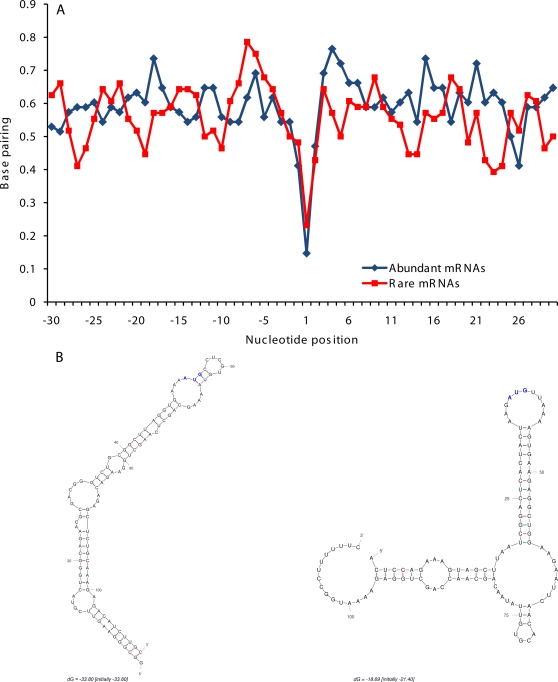
RNA secondary structures in the vicinity of the start codon. A. Profiles of nucleotide base pairing in the vicinity of the start codon for abundant and rare PK transcripts. Nucleotide positions are shown relative to translation start codon. B. Examples of predicted local secondary structures in the vicinity of the start codon for an abundant PK transcript (VRK1, NM_003384) and a rare PK transcript (MYLK4, NM_001012418). Start codons are shown in blue.

It was shown earlier that selection may be operating in the protein coding regions on most variable synonymous positions to maintain a more stable and ordered mRNA secondary structures [30]. To evaluate secondary structures in 5′UTRs, we computationally “folded” sequences of mature PK transcripts. In agreement with the results of transcriptome-wide analysis of mammalian mRNA folding, we found that secondary structures are frequently formed in PK transcripts between UTRs and CDSs in the vicinity of the start and stop codons (Figure S4). Sequences immediately upstream from the start codon have strong hybridization affinity to the N-terminal coding sequences, thus promoting formation of local hairpin structures where the start codon is positioned at the end of a hairpin in a relaxed loop (Figure 6). This type of secondary structure was facilitated by the enhanced GC-content in the N-terminal protein coding regions, and was observed in both abundant and rare transcripts. However, thermodynamic stability of these characteristic hairpin structures was significantly higher for abundant PK transcripts, than for rare transcripts (*p*<10^−5^).

Abundant PK transcripts also carry significantly more conserved 3′UTRs, relative to rare transcripts (*p*<10^−4^). No significant difference in nucleotide levels was observed between 3′UTR of abundant and rare PK transcripts, which had uniformly high AT content (Table 1).

### Nervous tissue-specific regulatory signals

**Figure 7:**
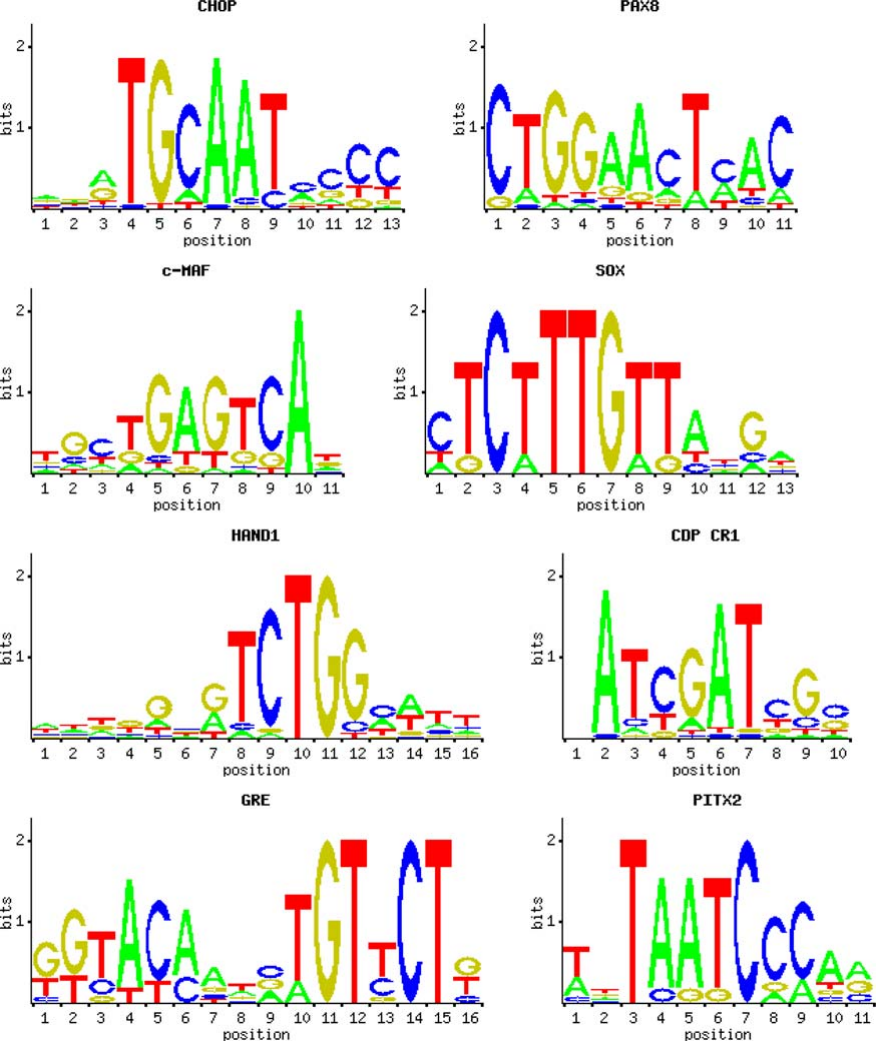
Characteristic transcription factor binding sites over-represented in evolutionarily conserved regions of PK genes predominantly expressed in nervous tissue.

PK genes up-regulated in the nervous tissue had significantly longer 5′-spacers and introns then genes down-regulated in the nervous tissue. These extended gene loci may harbor binding sites for nervous tissue-specific transcription factors. To identify brain-specific regulatory elements, we analyzed conserved synteny regions of PK genes predominantly expressed in the nervous tissue with the DiRE program. Whole gene loci, including promoters, UTRs, introns, and distant intergenic and spacer regions were included in this analysis. Conserved synteny regions of PK genes with similar overall expression levels and low expression in the brain tissue were used as a background set. Typical transcription factor binding sites overrepresented in evolutionarily conserved brain-specific PK genes are shown in Figure 7, and the top 30 overrepresented motifs are presented in Table S2. As seen from the Table, binding sites for POU, Pit, Pbx, Pax, Olf, Meis and other neuron-specific transcription factors that perform specific functions in the central nervous system were highly overrepresented in evolutionarily conserved in regions of PK genes predominantly expressed in the nervous tissue.

We searched for overrepresented conserved motifs in promoters of PK genes up-regulated in the nervous tissue using the DME program. Conserved promoter sequences of genes down-regulated in the nervous tissue were used as background in this analysis. Promoter regions of PK genes predominantly expressed in nervous tissue contained numerous over-represented sites that compositionally and textually differed from the sites associated with transcript abundance (data not shown). They were enriched with CTGG, TGCA, TCTGG, CAATC and CTGA motifs that constituted nucleotide core sequences for neuron-specific transcription factors identified with the DiRE program.

**Figure 8:**
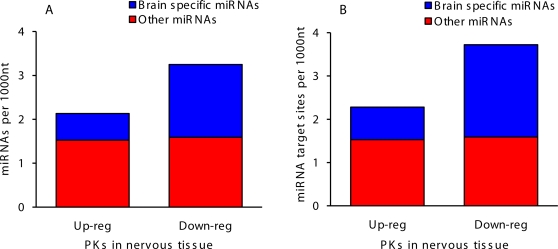
Hybridization affinity of human miRNAs to 3′UTRs of human PK genes up-regulated and down-regulated in nervous tissue. A. Predicted numbers of miRNAs hybridizing to 3′UTRs. B. Predicted numbers of miRNA target sites in 3′UTRs.

The number of predicted functional signals in 3′UTRs correlated with sequence conservation, indicating a significant level of evolutionary conserved posttranscriptional regulation in nervous tissue. To evaluate potential regulation of PK expression by RNA inhibition, we analyzed hybridization affinity of annotated human miRNAs to 3′UTRs of human PKs up-regulated and down-regulated in nervous tissue. Remarkably, we observed a significant difference in the number of binding sites for neuron-specific miRNAs between the two groups of PK transcripts (Figure 8). Transcripts rarely encountered in the nervous tissue were enriched 2–3 fold with binding sites for neuron-specific miRNAs, which likely facilitated targeted degradation of these transcripts in the nervous tissue through the RNA inhibition mechanism.

## Discussion

### Gene architecture and expression levels

Physiological complexity of an organism is largely dependent on the regulation of gene expression and correlates with the length of non-coding DNA, and the number of cis-control elements in genome [31]. It is estimated that as much as a third of the human genome controls chromosome replication, condensation, pairing, segregation, and gene expression [32]. The regulatory array of a typical mammalian gene locus consists of a core promoter, and proximal promoter elements (located within ∼3 kb in the upstream spacer region, 5′UTR and sometimes within the first exon of a gene), and distant enhancers, silencers, chromatin insulators, and scaffold/matrix attachment regions that can be scattered within 100 kb or more from the transcription start site. The size of a gene locus inversely correlates with GC content and depends on gene location within the complex landscape of the human genome, which is dominated by extensive GC-rich regions (isochores) alternating with GC-poor isochores. GC-rich isochores are densely populated with compact genes, while GC-poor isochores are low gene density regions populated with larger genes [33]. PK genes occupy larger genomic loci than other mammalian genes. Our results indicate that human and mouse PK genes expressed at different levels and in different tissues have similar GC-content in intergenic regions, suggesting that they are not confined to GC-rich isochores. Noteworthy, 5′-spacer regions and introns of the human PK genes were significantly (∼15%) longer than the corresponding regions of the mouse genes, implying a more complex regulation of transcription.

There is a strong negative correlation between gene length and the level of gene expression. Highly expressed human housekeeping genes are generally shorter and more compact than genes that are expressed at lower levels, which has been explained by selection pressure to reduce metabolic costs of transcription [24], [34]. Our results (Figure 2), consistent with published data for non-regulatory genes [34], [35] indicate that PK genes have significantly longer spacer regions, introns, primary transcripts, and encode larger and more complex proteins than average housekeeping and non-housekeeping mammalian genes. Within the PK group, we observed major differences in gene architecture that are related to gene expression levels and tissue-specific expression. Gene transcription levels and breadth of expression negatively correlate with the length of introns and the size of primary PK transcripts. Primary transcripts of highly and ubiquitously expressed genes are significantly shorter than primary transcripts of low expression PK genes. Average length of protein coding regions in ubiquitously expressed PK genes tends to be shorter than in other groups, which may reflect evolutionary pressure to reduce costs of translation for housekeeping protein kinases.

Transcription efficiency of a gene is determined by many factors, most important of which are the structure of promoter, the array of transcription factor binding sites, and the length of transcribed units. Mammalian transcription factor binding sites are usually organized in clusters that contain several phylogenetically conserved sites for a few different transcription factors [36]. Degenerate sites are enriched around the cognate binding sites in orthologous mammalian promoters [37], which may facilitate recruitment of transcription factors to DNA regulatory regions and a robust transcription response (for discussion of possible molecular mechanisms, see [37]). Our data show that GC content in promoter regions and 5′UTRs of ubiquitously expressed PK genes is stably higher, relative to other groups.

Proximal 5′-spacers of actively and ubiquitously transcribed PK genes carry complex cis-regulatory modules enriched with conserved and degenerate GC-rich binding sites for transcriptional activators of Sp, AP, and other families. Abundance of these sites is indicative of increased promoter strength and correlates with gene transcription levels.

### PK expression in nervous and testicular tissue

Human brain and testes employ large groups of tissue-specific PKs (Table S1), implying increased complexity of phosphorylation-dependent regulatory network that controls the functioning and homeostasis of these organs. We observed major differences in gene architecture and expression between PK groups up-regulated in nervous and testicular tissues. Adult brain is characterized with significant transcriptional and very low mitotic activity. Our data demonstrate that PK genes up-regulated in nervous tissue possess long transcribed regions with numerous extended introns, an indication of increased complexity of transcription regulation. They also carry extended spacer regions which may be required for accommodation of distant neuron-specific enhancers and extended 5′UTRs, an indication of increased post-transcriptional regulation.

In contrast, testis-specific PK genes are compact, with short and low conserved regulatory regions. These properties of testis-specific PK genes likely reflect simpler transcriptional control by testis-specific transcription factors that turn transcription on and off at specific stages during linear progression of germ cell development. Male germ cell development and differentiation require massive production of testis-specific transcripts and isoforms. Transcription during germ cell development peaks at meiotic and post-meiotic phases [38]. This increased expression fulfils the requirement for the rapid accumulation of large amount of transcripts in haploid round spermatids in preparation for radical restructuring of the cytoplasm and chromatin condensation at the following stage of elongated spermatids. After this final burst, transcription ceases in elongated spermatids, chromosomes are stripped of nucleosomes and densely packed in sperm heads. We speculate that general compactness of genomic loci of PK genes predominantly expressed in the testis [39], small size of their pre-mRNAs and reduced number of introns (Table 1) are likely dictated by the need for intense transcription in germ cells during the relatively short developmental time frame. Interestingly, several testicular PKs (TSSK1, TSSK2, SSTK and Haspin) are evolutionarily young, intronless, and supposedly originated by retrotransposition [7]. Same tendency for gene compactness was observed for proliferatively active placental tissue (data not shown).

### Evolutionary divergence of PK genes

Protein coding sequences of PK genes are evolutionarily more conserved than coding sequences of other genes (Table 2), which is likely due to tighter purifying selection on catalytic kinase domains. Evaluation of sequence divergence between the human and mouse PKs revealed differing rates of evolution for different gene groups. Ubiquitous PK genes evolve slower, relative to differentially expressed genes. Elevated conservation in ubiquitous PKs likely reflects increased selection pressure on housekeeping kinases. These data are in agreement with the observation that proteins with a broad range of tissue expression tend to be more conserved than those expressed in one or few cell types [26].

Our results demonstrate that genes preferentially expressed in different tissues evolve with differing rates. Rates of evolutionary divergence of PK genes up-regulated in nervous and testicular tissues correlated with the proliferative activity of the tissue and with the length of transcribed gene domains. Consistent with the published results [7], the group of testis-specific kinases is the most divergent between the human and mouse in the protein coding regions. This divergence is reflective of higher evolutionary rates of testicular PKs. Conversely, PK genes preferentially expressed in nervous tissue are more conserved between the human and mouse in the coding regions and UTRs, indicating elevated selection pressure on amino acid sequences and on RNA regulatory sequences.

Interestingly, our results demonstrate increased evolutionary divergence for the groups of PK genes with generally low expression levels and genes with low expression in the nervous tissue, relatively to ubiquitous PK genes. A likely explanation is that the low expression group contains genes selectively expressed in low abundant cell types. This group may also contain evolutionarily young genes with low expression levels and emerging function. Many of the genes from the second group are preferentially expressed in two or more tissues, suggesting possible diversification or specialization of function, which may be accompanied with evolutionary divergence. It is possible that some of the observed differences in sequence conservation between the human and mouse may be due to the unique physiology of the mouse. Consistent with published reports [40], [41], evolutionary conservation in the protein coding regions strongly correlated with conservation in 3′UTRs and 5′UTRs for all gene groups. These correlations were more pronounced for differentially expressed PK genes than for ubiquitously expressed genes. Our results for PK genes are in good agreement with published data for other genes [26] and support the idea that expression patterns affect selection intensity but not mutation rate.

### Regulation of expression by 3′UTRs

In eukaryotic cells, transcripts exist as complexes with associated proteins that are essential for mRNA transport across nuclear membrane, stability, and translation. Messenger RNA degradation and translation are tightly coupled events, and efficiency of gene expression is largely dependent on post-transcriptional stability of mRNA. Both mRNA stability and translation efficiency depend on the 3′ poly(A) tail which interacts with poly(A) binding protein (PABP). PABP is involved in mRNA circularization by binding together the 5′ and 3′ ends of mRNA, and also plays a role in translation initiation and mRNA degradation. The major pathway of eukaryotic mRNA decay is initiated with degradation of the 3′ poly(A) tail and the loss of PABP, linearization of transcript, and cleavage of the methylated 5′ cap structure, followed by 5′ to 3′ exonucleolytic degradation of mRNA (reviewed in [42]). Other RNA-binding proteins regulating post-transcriptional mRNA stability and turnover specifically interact with AU- and CU-rich sites in 3′UTRs [43]. For example, stability of the epidermal growth factor (EGF) receptor tyrosine kinase mRNA is mediated by two RNA-binding proteins that bind to AU-rich elements in the 3′UTR. The binding affinity of these proteins is down-regulated by the kinase ligand, EGF [44].

Another major regulatory pathway is RNA inhibition. 3′UTRs are recognized and targeted by small non-coding miRNAs that inhibit translation, promote transcript degradation, and often act as tissue-specific regulators of transcript stability [45]. A large set of housekeeping genes involved in basic cellular processes avoid regulation by miRNAs due to short 3′UTRs that are depleted of miRNA binding sites [46]. Our results are suggestive that PK genes are extensively regulated through RNA inhibition in a tissue-specific manner. 3′UTRs of transcripts rarely encountered in the nervous tissue were enriched with binding sites for neuron-specific miRNAs (Figure 8), which likely facilitate targeted degradation of these transcripts in the nervous tissues through the RNAi mechanism. Comparison of our results with published data [47] reveal that sites over-represented in the brain-specific PK transcripts include few ubiquitous miRNA binding sites that are commonly found in human transcripts, suggesting regulation by unidentified miRNAs. 3′UTRs of PK genes predominantly expressed in the nervous tissue were significantly longer and more conserved, as compared to genes expressed in the nervous tissue at low levels. These conserved GC-rich sites participate in the formation of local secondary structures in 3′UTRs which increase the compactness of transcript folding (data not shown) and may be involved in regulation of mRNA stability.

### Regulation of expression by 5′UTRs

Recent genetic studies demonstrated that mutations and single nucleotide polymorphism in 5′UTRs affect transcription efficiency, mRNA levels, and have implications in human disease [48], [49], [50], [51]. The length of the 5′UTR negatively correlates with mRNA and protein expression levels. Transcripts of highly expressed housekeeping genes carry short 5′UTRs devoid of strong secondary structures. Conversely, transcripts of low expression regulatory genes controlling cell proliferation, survival and apoptosis carry long 5′UTRs with stable secondary structures and upstream translation start codons [35], [52], [53]. Similar to other regulatory genes, PK genes possess long and complex 5′UTRs. Surprisingly, our analysis showed that abundant PK transcripts carry longer 5′UTRs with higher GC-content that form more stable secondary structures, as compared to rare PK transcripts. GC-rich elements in 5′UTRs are mostly confined to evolutionarily conserved sequences and could be maintained by selective pressure due to conserved biological function. Our observations suggest that GC-rich elements in 5′UTRs may function at the mRNA level rather than at the DNA level. Increased GC content in 5′UTRs of abundant transcripts allows formation of more stable RNA secondary structures that may serve as scaffolds for RNA-binding proteins, promote a more compact folding and increased mRNA stability.

The majority of translational control events occur at the level of initiation, implicating the 5′UTR as the major site of translational regulation. The cap-dependent initiation of translation is affected by mutations in the 5′UTR and severely hampered by stable secondary structures that can stall the ribosome and inhibit translation [54], [55]. Translation of mRNAs encoding regulatory proteins is often initiated via internal ribosome entry or other yet unknown mechanisms [56]. Transcript folding in the vicinity of the start codon favors formation of characteristic local structures where the start codon is positioned at the end of a hairpin in a relaxed loop [30]. This type of secondary structure is preferred in GC-rich 5′UTRs of abundant PK transcripts (Figure 6A) and probably represents adaptation for a more efficient translation of abundant mRNAs. Positioning of the AUG codon at the end of stem-loop may provide a more productive recognition context during cap-independent initiation of translation, when the ribosome binds directly to internal entry site without unwinding and scanning upstream regions of mRNA molecule.

A role was proposed for 5′UTRs in regulation of translation through intermolecular base-pairing interaction with 18S ribosomal RNA. It was hypothesized [57] and experimentally confirmed [58] that accessible “clinger” regions of 18S rRNA may function as low-specificity mRNA-binding sites allowing a more efficient transcript interaction with the ribosome. This interaction may affect translational efficiency of different subsets of mRNAs. Our data suggest that 5′UTRs of abundant PK transcripts have significantly higher hybridization affinity to 18S rRNA, than 5′UTRs of rare PK transcripts (Figure 5). Most common elements in 5′UTRs of abundant PK transcripts are short GGC repeats. CGGCGG element was recently identified as a core of a translation enhancer commonly occurring in 5′UTRs of mammalian mRNAs, which base pairs to a “clinger” site on 18S ribosomal RNA and facilitates translation initiation [29]. Studies of the functional role of CGG repeats in the 5′UTR of the human FMR1 gene demonstrated that these repeats may exert both positive and negative effects on the efficiency of translation of FMR1 mRNA, depending on repeat length. Long repeats in 5′UTR of FMR1 suppressed translation. However, the presence of short repeats increased translation efficiency in the absence of any change in mRNA levels [59]. Our results are consistent with these data and provide additional support for the ribosome filter hypothesis [58]. Other evolutionarily conserved GC-rich motifs in 5′UTRs of abundant PK transcripts (Table S1) may affect translation initiation through similar mechanisms.

### Conclusions

Genomic organization of the human PK superfamily and the structure of gene functional domains significantly differ from other protein coding genes. Our results demonstrate that gene expression levels, expression breadth, and requirements for tissue-specific regulation correlate with genomic architecture. These factors also may contribute to selection pressure in the protein-coding and non-coding DNA regions. Transcription levels and breadth of expression negatively correlate with the length of introns and the size of primary transcript, which is likely due to the necessity to minimize metabolic costs of transcription for abundant mRNAs. It is generally accepted that mammalian ubiquitously expressed genes evolve slower than tissue-specific genes. Here we show that genes up-regulated in different tissues evolve with different rates, and that evolutionary rates correlate with the proliferative activity of expressing tissue and with gene architecture. The observed negative correlation between the length of transcribed gene domains and the proliferative activity of tissues may reflect metabolic constraints and requirements for tissue-specific expression. Our data provide evidence that evolutionarily conserved phylogenetic footprints and structural elements in messenger RNA play roles in regulation of transcript abundance, tissue-specific expression, and translation. All these mechanisms may contribute to the multi-level regulation of PK expression, providing precision control of the key components of the cell signaling pathways that determine cell function and destiny.

## Methods

### Gene sequences and alignments

Protein kinase names and classification in this paper are presented according to Manning et al [5]. Sequences of the full-length human PK mRNAs were downloaded from http://kinase.com/kinbase/FastaFiles/Human_kinase_rna.fasta and aligned to the sequence of the human genome (ftp://hgdownload.cse.ucsc.edu/goldenPath/hg18/chromosomes/), March 2006 assembly. To compare our results for PK genes with overall trends for other genes, we compiled control group of 7,711 randomly selected non-PK genes well-annotated orthologous human and mouse genes that yielded high quality genome alignments. Gene coordinates were downloaded from http://genome.brc.mcw.edu/cgi-bin/hgTables. For each human mRNA, a mouse orthologue was found as a best Blast hit with complete mouse mRNA sequences. Only full-length transcripts with links to the RefSeq database were used (http://www.ncbi.nlm.nih.gov/RefSeq/index.html). Mouse genome sequences (February 2006 assembly) were downloaded from ftp://hgdownload.cse.ucsc.edu/goldenPath/mm8/chromosomes/. Genome coordinates of extended human gene loci were transferred to the mouse genome sequence with UCSC Lift Genome Annotations tool (http://genome.brc.mcw.edu/cgi-bin/hgLiftOver). Mammalian genomic repeats were masked and extended genomic loci of orthologous human-mouse genes were aligned with the OWEN program [60] and annotated. In case of alternatively spliced forms, the longest CDSs and UTRs were considered. For the protein coding regions, the alignment of nucleotide sequences was guided by the amino acid sequence alignment. 5′ and 3′ intergenic regions were considered separately and orientations of gene loci were assigned from the 5′ end to the 3′ end of a gene. For 98% of PK genes, UTRs were annotated and considered separately. When UTRs are included in the intergenic regions, they usually constitute only a minor fraction of the sequences and can not affect significantly the results of computer analysis of intergenic regions. We additionally analyzed proximal 5′-spacer regions (3 kb upstream from the transcription start site) that contain core promoter and proximal promoter elements. Overall level of similarity was calculated from percent identity in global alignments and sequence lengths. Core hits with *e*-value lower than 10^−3^ produced by OWEN program were extracted for analysis. Phylogenetic footprints were identified using conservative parameters (match = 1, mismatch = −1, gap = −5/−1) where the final similarity of the extended core regions was higher than 50% and boundaries were matches. The lowest significantly non-random level of similarity for two-sequence alignments (A ∼30%, T ∼30%, G ∼20%, C ∼20%) is 42% [61]. Statistical analyses of phylogenetic footprints were conducted using Excel (Microsoft, USA) and our statistical tools [62].

### Evaluation of gene expression levels

We evaluated relative transcript abundance using the numbers of gene-specific expressed sequence tag (EST) sequences in GenBank. We used EST approach because it allows a more reliable identification of the transcript identity than microarray data and has a greater potential for quantitative analysis, since EST clone frequency in a library is generally proportional to the corresponding gene expression levels. This approach gives a reasonably accurate approximation of gene expression and was successfully used for studying gene transcription levels and tissue-specific gene expression (for example, [24], [26], [63], [64], [65]). We aligned sequences of PK mRNAs with PK-specific ESTs from the human normal tissue EST libraries from GenBank using the program BLAST [66]. We accepted EST hits with the identity more than 95% and longer than 80% of EST sequence length as matches. For identification of PKs with overall high and low expression levels we selected genes with EST numbers >140 and <25, correspondingly, from pooled normal tissue EST libraries. For identification of PKs up-regulated and down-regulated in tissues, we normalized each EST library to the same level using published approach [63], [65]. We calculated PK tissue expression score (ES) as the ratio of PK-specific ESTs versus expected EST frequency. We considered PKs with ES>6 (ES>2 for brain and nervous tissue) as preferentially expressed in a tissue. PKs with ES<0.25 from nervous tissue or testis were considered as down-regulated in these organs. Breadth of gene expression was estimated as the number of organ and tissue sources of gene-specific ESTs. For identification of the group of ubiquitously expressed PKs, we used tissue EST libraries containing more than 100,000 ESTs. Genes with low expression levels and low EST numbers, which could not be reliable evaluated by this method, were excluded from this analysis. Genes were ranked according to the number of expressing tissues. Genes expressed in 9 or more tissues from the 12 tissues were considered as broadly expressed. Groups of preferentially expressed PK genes were checked against the Gene Expression Atlas (http://wombat.gnf.org/) and available experimental PT-PCR and Northern data from literature. Only genes with similar tissue-specific preferences were considered in the final classification and computer analysis.

### Textual and statistical analyses

Human-mouse evolutionary divergence of PK genes was evaluated using Kimura's two parameter model [28]. The levels of synonymous and non-synonymous divergence (K_s_ and K_a_, respectively) were calculated with the PAML program (ftp://abacus.gene.ucl.ac.uk/pub/paml) using default parameters and the yn00 estimation method [25]. For all measures of evolutionary distances, including K_s_, K_a_, K_a_/K_s_, the Wilcoxon rank sum non-parametric test was applied to the pairwise comparison between all groups of PK genes.

To identify regulatory elements associated with transcript abundance and tissue-specific expression, we searched for conserved over-represented motifs in promoter regions of actively transcribed genes using the discriminating matrix emulator (DME) program [67]. Search for over-represented sequence elements in 5′UTR and 3′UTR regions was performed using an enumerative Markov chain motif finding algorithm [68], which applies *z*-scores to evaluate the over-representation of exact DNA words, and SiteDB program [69]. We also used the program CLOVER [70] that uses the position frequency matrices (PFMs) of cis-regulatory sites to evaluate sequences for statistically significant over/under-representative sequence elements. The methods employed take into account nucleotide content bias. Identified statistically significant over-represented motifs were compared with PFMs of known cis-regulatory motifs from the TRANSFAC database (http://www.biobase-international.com/pages/index.php?id=transfac) [71]. DiRE server for the identification of distant regulatory elements of co-regulated genes (http://dire.dcode.org) was used for prediction of transcription factor binding sites over-represented in conserved synteny regions of PK genes predominantly expressed in nervous tissue [39].

Formation of intermolecular mRNA-rRNA duplexes and hybridization affinity of 5′UTRs to ribosomal RNA were evaluated with program Hybrid [62] under default parameters using ΔG threshold of ≤−17 kcal/mol [57]. Annotated dataset of 476 human miRNAs was extracted from Rfam database, release10 (http://microrna.sanger.ac.uk/sequences/index.shtml). For identification of potential miRNA target sites in 3′UTRs, we calculated hybridization affinity of miRNAs to 3′UTRs using Hybrid program and ΔG threshold of ≤−17 kcal/mol, and used predictions of RegRNA program (http://regrna.mbc.nctu.edu.tw/). For identification of potential binding sites for neuron-specific miRNAs in 3′UTRs, we calculated hybridization affinity of 3′UTRs to annotated neuron-specific and brain-specific miRNAs from Rfam database. We identified common invariant oligonucleotides in 3′UTRs. We required common fragments of complementarity to be at least 6 nt long, since most identifies targets have conserved complementary seeds of 6–8 nucleotides. We performed single-linking clustering of these targets using the Histogram AC program [69].

Statistical analysis was performed using exact Fisher test, Student's t-test for normally distributed variables, Wilcoxon rank sum test for unknown distributions.

## Supporting Information

Table S1 PK-specific ESTs in GenBank originating from different human organs. Differentially expressed PK genes. Evolutionarily conserved motifs over-represented in promoter regions and 5′UTRs of high expression PK genes.
(0.23 MB XLS)

Table S2 Top 25 transcription factor binding sites over-represented in evolutionarily conserved regions of PK genes preferentially expressed in the nervous tissue.
(0.04 MB DOC)

Figure S1 Length of functional domains in the groups of differentially expressed human and mouse PK genes. A. Length of 5′-spacers, introns, and primary transcripts. B. Length of CDSs, 5′UTRs and 3′UTRs.
(0.57 MB TIF)

Figure S2 Correlation between PK expression levels and rates of non-synonymous human-mouse evolutionary divergence (Ka). Gene expression levels were estimated as the number of gene-specific ESTs in GenBank.
(0.34 MB TIF)

Figure S3 Characteristic evolutionarily conserved motifs over-represented in promoter regions of high expression PK genes.
(1.38 MB TIF)

Figure S4 Profiles of nucleotide base pairing in PK transcripts around the start codon (A) and the stop codon (B) with different mRNA structural domains. Blue, nucleotides paired with the 5′-UTRs; red, nucleotides paired with the CDSs; green, nucleotides paired with the 3′-UTRs; black, total base paired nucleotides.
(0.88 MB TIF)
