# Supplementary material for: Expression Patterns of Protein Kinases Correlate with Gene Architecture and Evolutionary Rates
Source: PLoS One. 2008 Oct 31;3(10):e3599. doi: 10.1371/journal.pone.0003599 (PMC2572838; doi:10.1371/journal.pone.0003599)
Supplement: Table S2 — Top 25 transcription factor binding sites over-represented in evolutionarily conserved regions of PK genes preferentially expressed in the nervous tissue. (0.04 MB DOC) [file pone.0003599.s002.doc]

**Table S2. Top 25 transcription factor binding sites over-represented in evolutionarily conserved regions of PK genes preferentially expressed in the nervous tissue.**

No Transcription Binding site Importance Function in

factor nervous tissue

1 CHOP NNRTGCAATMCCC 1.395 [1]

2 ZTA TNACNNTKRCWCA 1.337

3 PAX8 CTGGAACTMAC 1.105 [2]

4 c-MAF NGCTGAGTCAN 1.059 [3]

5 SOX CTCTTTGTTANGA 1.047 [4]

6 HAND1 NNNNGNRTCTGGMWTT 0.981 [5]

7 CDP CR1 NATCGATCGS 0.930 [6]

8 GRE GGTACAANNTGTYCTK 0.872 [7]

9 CDP CR3 CACCRATANNTATBG 0.814 [6]

10 PITX2 WNTAATCCCAR 0.756 [8]

11 POU6F1 GCATAAWTTAT 0.756 [9]

12 STAT5b NAWTTCYNGGAAWTN 0.755 [10]

13 Olf1 NNCDABTCCCYAGRGARBNKGN 0.640 [11]

14 Cdc5 GATTTAACATAA 0.640

15 Meis2 NNNTGACAGNNN 0.639 [12]

16 Brn2 TTATGYTAAT 0.569 [13]

17 AP4 RNCAGCTGC 0.560

18 Pbx GATTGATKGNNS 0.523 [12]

19 v-MAF NGCTGAGTCAN 0.515 [14]

20 CHX10 NNNTAATTAGCNNN 0.514 [15]

21 POU3F2 TTATGYTAAT 0.499 [16]

22 HOXA7 YCAATCT 0.465 [17]

23 FOXM1 ARATKGAST 0.465 [18]

24 PIT1 NMTTCATAAWTATWNMNA 0.465 [19]

25 NKX22 TTAAGTRSTT 0.465 [15]

Over-represented sites were identified with DiRE program (http://dire.dcode.org). Conserved sequences of PK genes expressed in nervous tissue at low levels were used as a background set in this analysis.

**REFERENCES**

1. Reimertz, C., et al., *Gene expression during ER stress-induced apoptosis in neurons: induction of the BH3-only protein Bbc3/PUMA and activation of the mitochondrial apoptosis pathway.* J. Cell. Biol., 2003. **162**(4): p. 11.

2. Quignodon, L., et al., *A combined approach identifies a limited number of new thyroid hormone target genes in post-natal mouse cerebellum.* J. Mol. Endocrinol., 2007. **39**: p. 17-28.

3. Kurschner, C. and J.I. Morgan, *The maf proto-oncogene stimulates transcription from multiple sites in a promoter that directs Purkinje neuron-specific gene expression.* Mol. Cell. Biol., 1995. **15**: p. 246-354.

4. Kuhlbrodt, K., et al., *Cooperative function of POU proteins and SOX proteins in glial cells.* J. Biol. Chem., 1998. **273**: p. 16050-16057.

5. Firulli, A.B., *A HANDful of questions: the molecular biology of the heart and neural crest derivatives (HAND)-subclass of basic helix-loop-helix transcription factors.* Gene, 2003. **312**: p. 27-40.

6. Uemura, O., et al., *Comparative functional genomics revealed conservation and diversification of three enhancers of the isl1 gene for motor and sensory neuron-specific expression.* Dev Biol., 2005. **278**: p. 587-606.

7. Kitchener, P., et al., *Differences between brain structures in nuclear translocation and DNA binding of the glucocorticoid receptor during stress and the circadian cycle.* Eur. J. Neurosci.,, 2004. **19**: p. 1837-1846.

8. Martin, D.M., et al., *PITX2 is required for normal development of neurons in the mouse subthalamic nucleus and midbrain.* Dev. Biol., 2004. **267**: p. 93-108.

9. Palm, K., et al., *Fetal and adult human CNS stem cells have similar molecular characteristics and developmental potential.* Mol. Brain Res., 2000. **78**: p. 192-195.

10. Bennett, E., et al., *Hypothalamic STAT proteins: regulation of somatostatin neurones by growth hormone via STAT5b.* J. Neuroendocrinol., 2005. **17**: p. 186-194.

11. Wang, S.S., et al., *Genetic disruptions of O/E2 and O/E3 genes reveal involvement in olfactory receptor neuron projection.* Development, 2004. **131**: p. 1377-1388.

12. Shim, S., et al., *Regulation of EphA8 gene expression by TALE homeobox transcription factors during development of the mesencephalon.* Mol. Cell. Biol., 2007. **27**: p. 1614-1630.

13. Castro, D.S., et al., *Proneural bHLH and Brn proteins coregulate a neurogenic program through cooperative binding to a conserved DNA motif.* Dev. Cell., 2006. **11**: p. 831-844.

14. Matsushima-Hibiya, Y., S. Nishi, and M. Sakai, *Rat maf-related factors: the specificities of DNA binding and heterodimer formation.* Biochem. Biophys. Res. Commun., 1998. **245**: p. 412-418.

15. Briscoe, J., et al., *A homeodomain protein code specifies progenitor cell identity and neuronal fate in the ventral neural tube.* Cell, 2000. **101**: p. 435-445.

16. Josephson, R., et al., *McKay, R.D. POU transcription factors control expression of CNS stem cell-specific genes.* Development, 1998. **125**: p. 3087-3100.

17. Dasen, J., et al., *A Hox Regulatory Network Establishes Motor Neuron Pool Identity and Target-Muscle Connectivity.* Cell, 2005. **123**: p. 477-491.

18. Schüller, U., et al., *Forkhead transcription factor FoxM1 regulates mitotic entry and prevents spindle defects in cerebellar granule neuron precursors.* Mol. Cell. Biol., 2007. **27**: p. 8259-8270.

19. Ingraham, H.A., et al., *A Family of Pou-Domain and Pit-1 Tissue-Specific Transcription Factors in Pituitary and Neuroendocrine Development.* Ann. Rev. Physiol., 1990. **52**: p. 773-791.
