# Supplementary figures and images for: Expression Patterns of Protein Kinases Correlate with Gene Architecture and Evolutionary Rates
Source: PLoS One. 2008 Oct 31;3(10):e3599. doi: 10.1371/journal.pone.0003599 (PMC2572838; doi:10.1371/journal.pone.0003599)

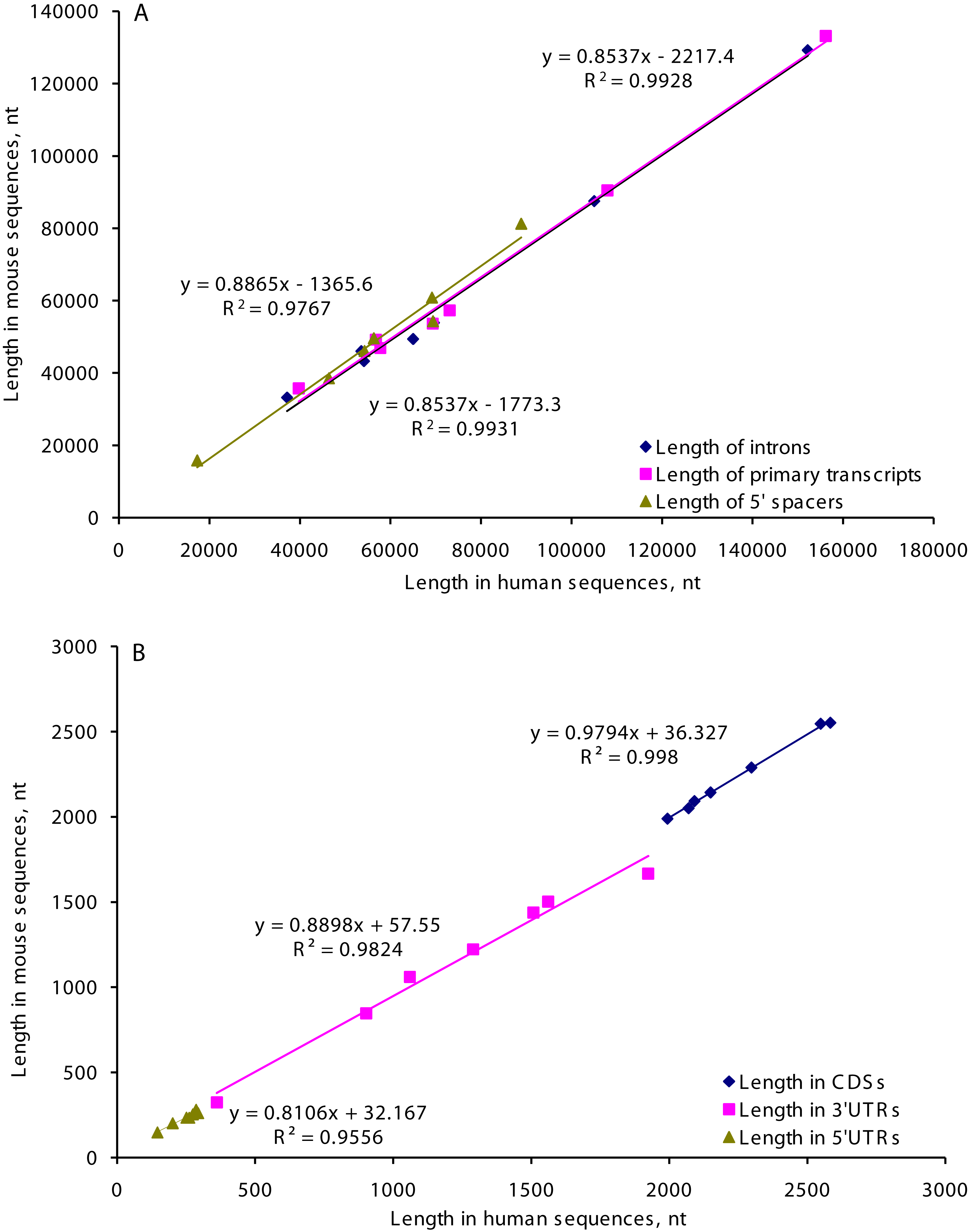

Supplement: Figure S1 — Length of functional domains in the groups of differentially expressed human and mouse PK genes. A. Length of 5′-spacers, introns, and primary transcripts. B. Length of CDSs, 5′UTRs and 3′UTRs. (0.57 MB TIF) [file pone.0003599.s003.tif]

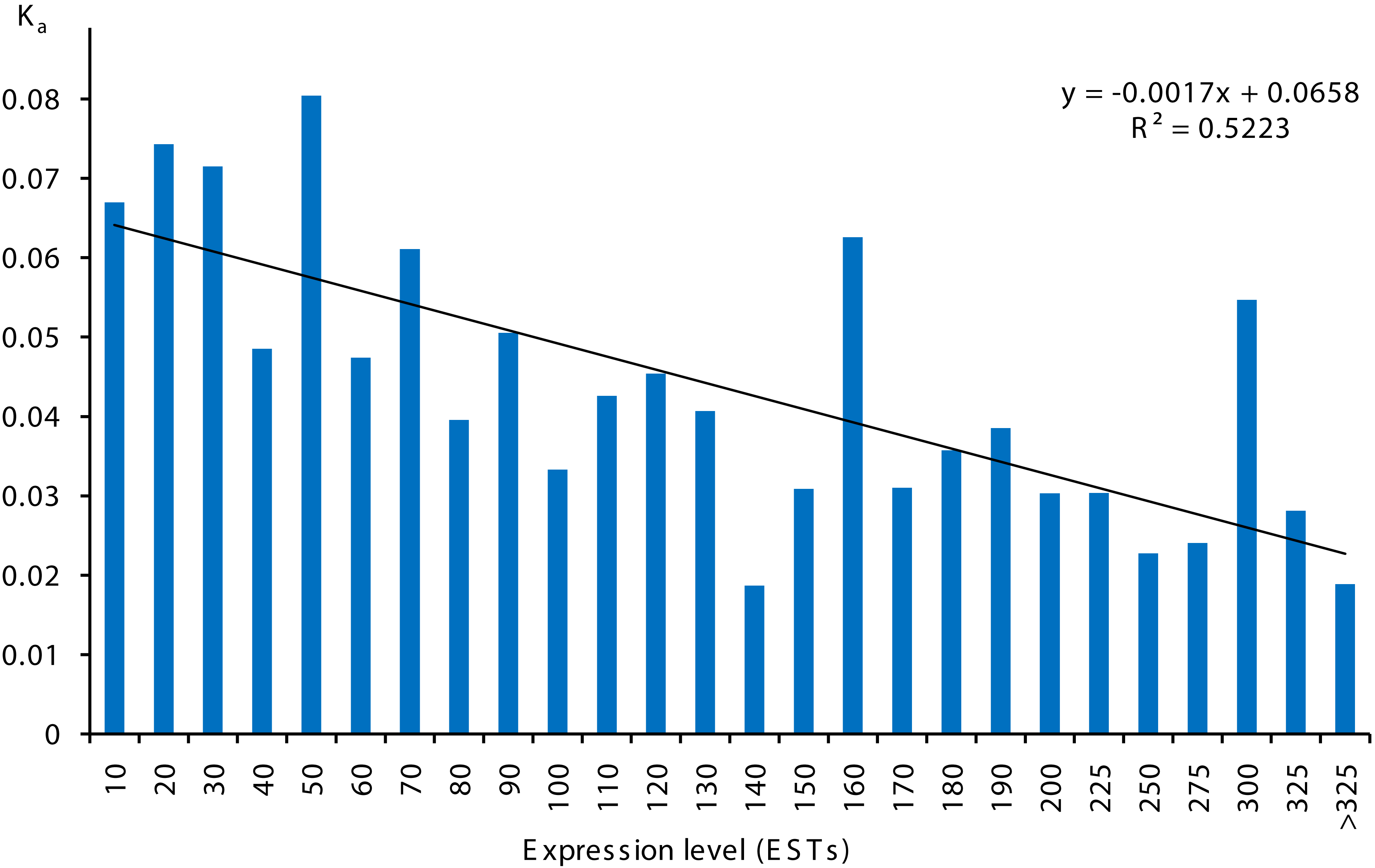

Supplement: Figure S2 — Correlation between PK expression levels and rates of non-synonymous human-mouse evolutionary divergence (Ka). Gene expression levels were estimated as the number of gene-specific ESTs in GenBank. (0.34 MB TIF) [file pone.0003599.s004.tif]

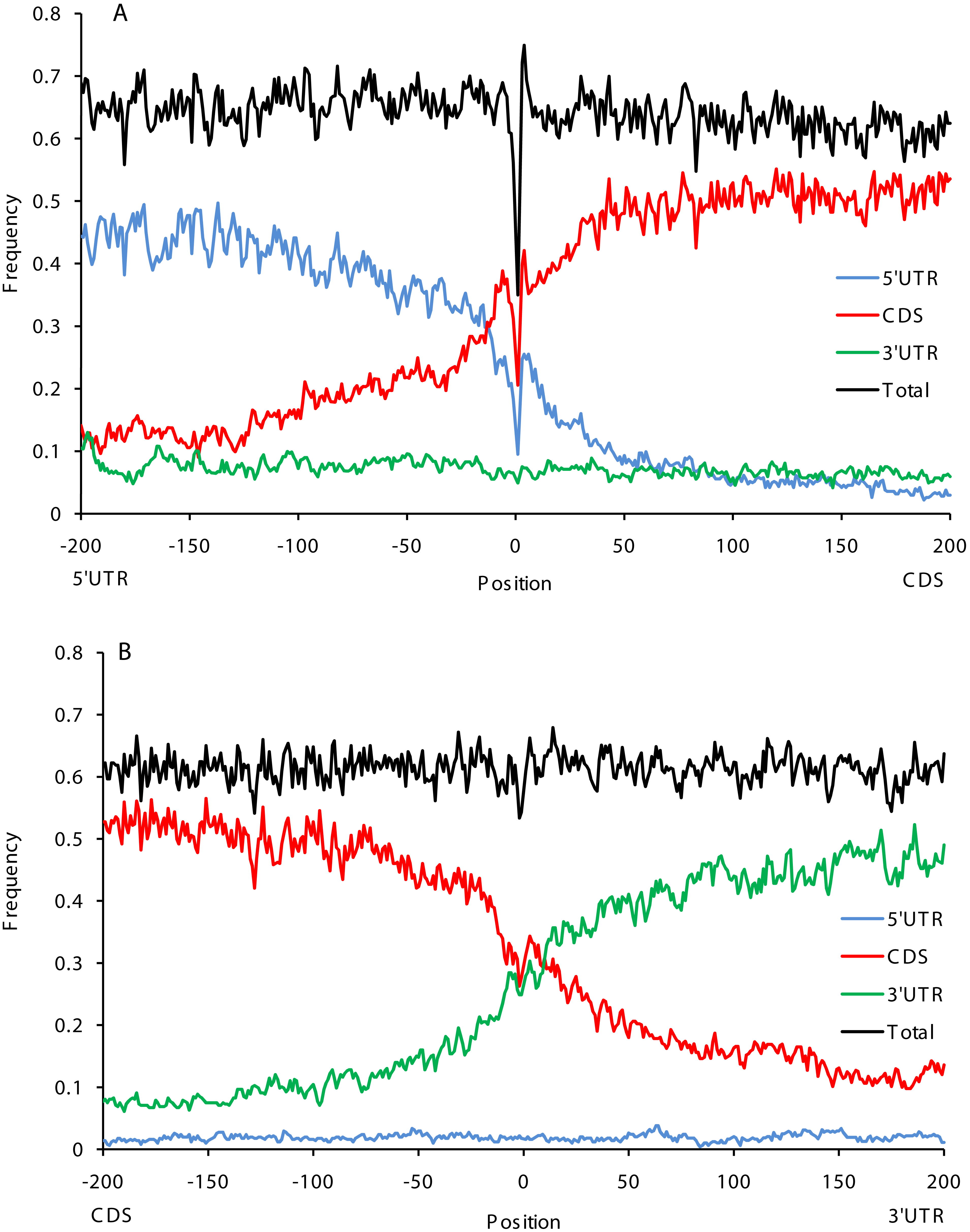

Supplement: Figure S3 — Characteristic evolutionarily conserved motifs over-represented in promoter regions of high expression PK genes. (1.38 MB TIF) [file pone.0003599.s005.tif]

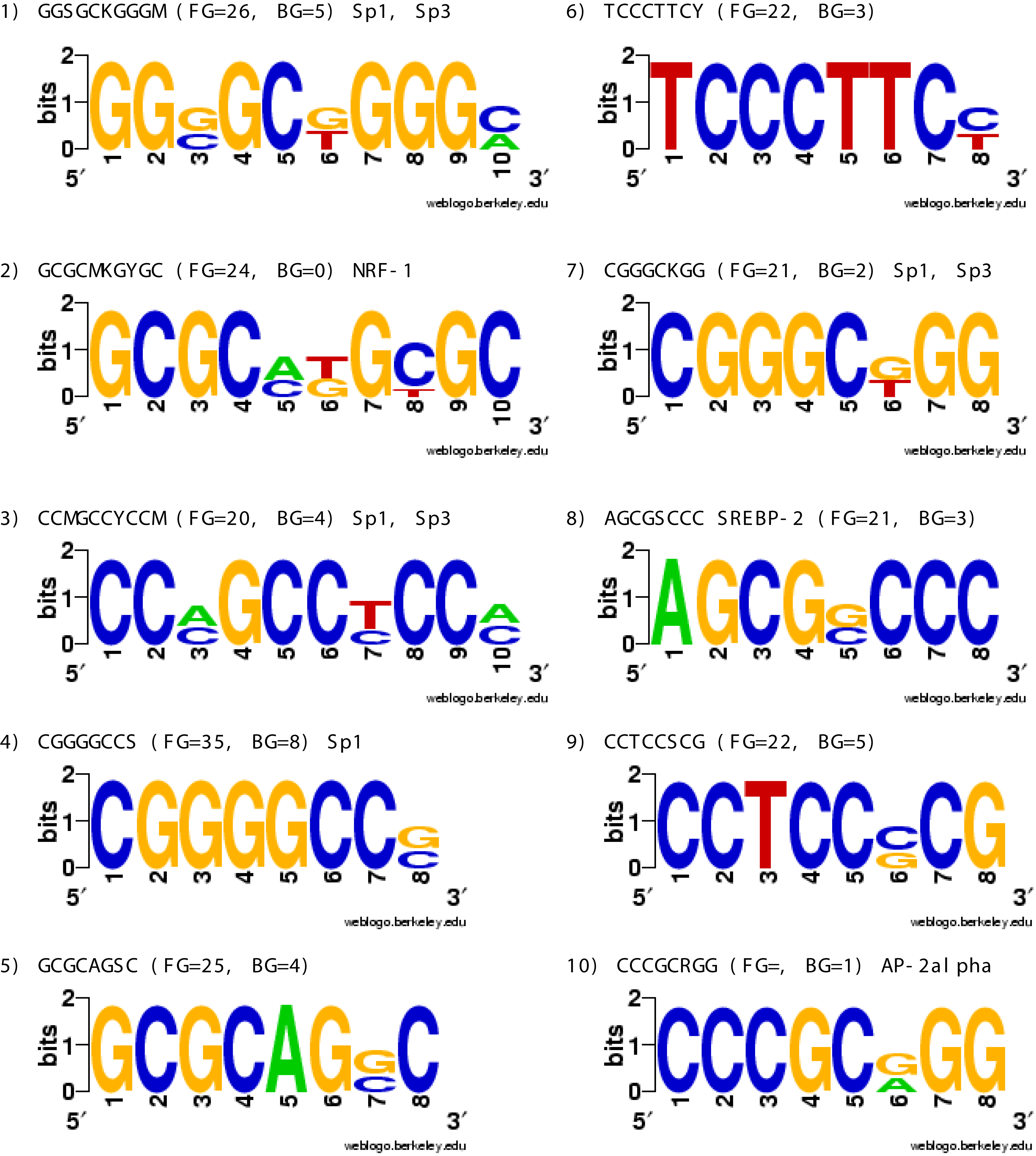

Supplement: Figure S4 — Profiles of nucleotide base pairing in PK transcripts around the start codon (A) and the stop codon (B) with different mRNA structural domains. Blue, nucleotides paired with the 5′-UTRs; red, nucleotides paired with the CDSs; green, nucleotides paired with the 3′-UTRs; black, total base paired nucleotides. (0.88 MB TIF) [file pone.0003599.s006.tif]
